# Childhood fever in well-child clinics: a focus group study among doctors and nurses

**DOI:** 10.1186/s12913-016-1488-1

**Published:** 2016-07-08

**Authors:** Kirsten K. B. Peetoom, Luc J. L. Ploum, Jacqueline J. M. Smits, Nicky S. J. Halbach, Geert-Jan Dinant, Jochen W. L. Cals

**Affiliations:** Department of Family Medicine, CAPHRI School for Public Health and Primary Care, Maastricht University, PO Box 616, 6200 MD Maastricht, The Netherlands; Envida homecare organisation, PO Box 241, 6200 AE Maastricht, The Netherlands

**Keywords:** Fever, Child, Information provision, Education, Well-child care, Preventive child healthcare

## Abstract

**Background:**

Fever is common in children aged 0-4 years old and often leads to parental worries and in turn, high use of healthcare services. Educating parents may have beneficial effects on their sense of coping and fever management. Most parents receive information when their child is ill but it might be more desirable to educate parents in the setting of well-child clinics prior to their child becoming ill, in order to prepare parents for future illness management. This study aims to explore experiences of well-child clinic professionals when dealing with childhood fever and current practices of fever information provision to identify starting points for future interventions.

**Methods:**

We held four focus group discussions based on naturalistic enquiry among 22 well-child clinic professionals. Data was analysed using the constant comparative technique.

**Results:**

Well-child clinic professionals regularly received questions from parents about childhood fever and felt that parental worries were the major driving factor behind these contacts. These worries were assumed to be driven by: (1) lack of knowledge (2) experiences with fever (3) educational level and size social network (4) inconsistencies in paracetamol administration advice among healthcare professionals. Well-child clinic professionals perceive current information provision as limited and stated a need for improvement. For example, information should be consistent, easy to find and understand.

**Conclusions:**

Fever-related questions are common in well-child care and professionals perceive that most of the workload is driven by parental worries. The focus group discussions revealed a desire to optimise the current limited information provision for childhood fever. Future interventions aimed at improving information provision for fever in well-child clinics should consider parental level of knowledge, experience, educational level and social network and inconsistencies among healthcare providers. Future fever information provision should focus on improving fever management and practical skills.

**Electronic supplementary material:**

The online version of this article (doi:10.1186/s12913-016-1488-1) contains supplementary material, which is available to authorized users.

## Background

Fever is common in children aged 0 to 4 years [1–3]. Parents’ misconceptions and unrealistic concerns make them easily worried when their child has a fever [4, 5]. They worry that fever might result in serious harm such as febrile seizures, brain damage or other adverse consequences [4, 6–9]. Subsequently, these worries drive healthcare-seeking behaviour. Schmitt introduced the term “fever phobia” in 1980 to describe these unrealistic fears in parents [5]. Since then, multiple studies have shown that fever phobia is still present [4, 9–14]. Important drivers of fever phobia and consultations to the general practitioner (GP) are a lack of parental knowledge on coping with fever but also parents’ perceived sense of control in the face of the perceived threat of an illness [5, 9, 15, 16].

Qualitative research in primary care has shown that there is a need to educate parents about fever and to influence parental perceptions of the threat posed by fever. Educating parents may enhance their self-management and sense of control, reduce healthcare-seeking behaviour and lower antibiotic prescriptions [9, 16, 17]. Most parents receive information at the time their child is ill but it might be more desirable to educate parents prior to their child becoming ill, in order to prepare parents for future illness management. The setting of well-child clinics (WCC) might be a good place to achieve this [12, 16–18].

WCC professionals encounter young children regularly for check-ups and information provision is an important pillar of well-child consultations [18, 19]. In the Netherlands approximately 95 % of children aged 0 to 4 visit the WCC for regular check-ups [20]. Parents tend to rely on WCC nurses for fever management advice and whether they should consult the GP or go to the hospital [21]. To illustrate, about one-third of 80,000 referrals to the GP by WCC professionals is for childhood infections [22]. Earlier studies have demonstrated that educating parents about childhood fever in the WCC setting may improve parental knowledge, attitude and use of healthcare services [23–32]. There are as yet no studies providing insight into the experiences and needs of WCC professionals in childhood fever consultations and WCC professionals’ current information provision practices.

The purpose of this study was to explore well-child care professionals’ experiences with childhood fever and current information provision practices when communicating with parents about fever management. By doing so, we hope to uncover barriers to and facilitators of information provision before a child’s new fever episode.

## Methods

This descriptive qualitative study was based on naturalistic inquiry. Naturalistic inquiry studies real-world phenomena in natural settings and aims to inductively construct theories by means of a continuous interplay between data collection and analysis. Naturalistic inquiry includes special criteria for trustworthiness [33].

### Study setting

Dutch youth healthcare focuses on providing preventive care services to children aged 0 to 19 years old. Children are seen for regular check-ups and vaccination jabs by specially trained doctors and nurses. WCC professionals concentrate on children aged 0 to 4 years old and see the children about 12 times. WCC professionals monitor the child’s health status and development, screen for congenital disorders, provide vaccination jabs, provide parents with information and counselling and coordinate healthcare needs. In the Netherlands approximately 95 % of the children aged 0 to 4 years old visit the WCC for regular check-ups [20]. Children are seen more frequently when there is need for extra care.

### Participants

We selected WCC professionals providing care to children aged 0 to 4 years in the Maastricht region of the Netherlands. By doing so, we covered neighbourhoods with varying socioeconomic statuses and educational levels. A staff physician of the WCC organization emailed all 30 WCC doctors and nurses to inform them about the study and invite them to participate. Both doctors and nurses were purposefully sampled to participate in focus group discussions. We aimed to mix doctors and nurses as they work in teams at the WCC and we wanted to facilitate and foster discussion among participants by including different viewpoints and experiences. We aimed to include 4–6 participants per focus group.

### Data collection

Focus groups (FG) were organised in November 2014 in Maastricht. The meetings lasted for 60 min each. The topic list was based on sensitizing concepts derived from scientific literature and expert discussions [34]. The topic list incorporated two main themes: (1) current experiences with childhood fever information provision to parents, and (2) exploration of starting points to improve future fever information provision (see Additional file 1). An independent and experienced moderator with a background as medical doctor led the focus group discussions. The moderator was accompanied by two observers (JS, LP) who are also MD, and the main researcher (KP) with a background in health sciences. The observers and main researcher made field notes during data collection. The focus group discussions were audio recorded and recorded on camera. We reached data saturation after three focus group discussions and performed one additional focus group to confirm the findings. JS and LP transcribed the focus group discussions verbatim and double-checked the transcripts.

### Data analysis

Data collection and analysis was performed simultaneously according to constant comparative technique [35]. Categories emerged inductively from the focus group data by performing open and axial coding, and deductively through constant comparison. The topic list was discussed and adjusted among the wider research team after every focus group discussion. The data was coded by three researchers (JS, LP, KP) with NVivo version 9. We discussed the codes, main categories and subcategories extensively in the research team and inconsistencies were resolved by consensus.

### Trustworthiness of data

To achieve methodological triangulation we combined focus group discussions and field notes. Next, to accomplish data source triangulation we incorporated experiences of WCC professionals who worked at different WCC locations within different neighbourhoods with a variety of socioeconomic statuses and educational levels. Furthermore, we assembled doctors and nurses together in focus group discussions to foster discussion from different professional viewpoints. Investigator triangulation was accomplished by incorporating researchers from different backgrounds in the research team, i.e. a health scientist, two medical doctors and a general practitioner. These were all actively involved in all stages of the research process. Peer debriefing meetings were held regularly among the research team. We performed a member check of the transcript among all participants [36, 37].

## Results

### Sample characteristics

A total of 22 well-child clinic professionals (seven doctors, 15 nurses) participated in four focus group discussions. The average age of participants was 45 years old and 21 were female. Average work experience at the WCC was 16.4 years, ranging from 0 to 36 years.

### Main category and subcategories

The coding process yielded one main category and four subcategories. Parental worries about childhood fever emerged as the main category from the data and was seen as the major driving factor behind most fever-related contacts with the WCC. We identified 4 subcategories which are all closely related to and possible drivers of parental worries: (1) level of knowledge of parents, (2) level of experience of parents, (3) influence of educational level and social network of parents and, (4) perceived degree of inconsistency in received advice. Together with the participants we explored starting points to improve current fever information provision.

### Parental worries

According to WCC professionals parents regularly contact them because they are worried about their child’s fever. These questions mostly focus on the degree of fever, cause and possible consequences of fever and their uncertainty regarding self-management strategies.

*“In particular when the temperature keeps on rising and reaches 40.2 °C, then parents start panicking, [saying] ‘Help, what should I do?’” (Nurse 3, FG3).*

WCC professionals generally feel that by asking questions, parents are seeking reassurance and to double-check if their actions and worries are legitimate.

*They just want to be heard. Or want to hear the opinion of a professional (Nurse 3, FG2).(…) Yes, they say they feel more reassured afterwards (Nurse 1, FG2). Because they know what steps to take (Nurse 4, FG2).*

WCC professionals believe that parents tend to be very fixated on the child’s temperature and are quickly worried, even when the body temperature is only slightly elevated, and overestimate the significance of an elevated body temperature as a single symptom.

*Parents’ worries often start as soon as the child’s temperature hits 37.8 °C and they’re like, “Oooohh” (Doctor 2, FG4).*

WCC professionals also expressed that according to them many parents fear that the raised body temperature reflects the severity of what is causing the fever. This is reinforced by previous negative experiences and stories from people in their surroundings.

*(…)They fear febrile seizures (Nurse 1, FG3).(…) Dehydration, meningitis (Nurse 2, FG3). And spots as well (Doctor 1, FG3).*

WCC professionals perceive their clinic as easily approachable and think that as a consequence parents typically do not hesitate to contact them, including for fever-related questions. This accessibility is highly esteemed by WCC professionals because they aim to empower parents in bringing up their children. However, they also acknowledged this approachability may also wrongly reinforce help-seeking behaviour. WCC professionals also find that parents contact them because they do not feel understood or taken seriously by their own GP and hope to find reassurance by asking questions of WCC professionals.

*Their GP has said so many times there is nothing wrong with their child. And then they call us (Doctor 2, FG1).*

### Level of knowledge

WCC professionals perceived that one of the major drivers of parental worries in childhood fever to be a general lack of parental knowledge regarding the definition of fever, fever pathophysiology and fever self-management strategies.

*Parents call and ask: “His temperature is slightly elevated, what to do? How long do we have to wait, should we give medication, is this normal? (…) My child has 37.6 degrees Celsius; is that a fever?” (Nurse 5, FG2).*

In the opinion of WCC professionals parents tend to ignore other symptoms of illness while WCC professionals themselves base their advice on the child’s overall presentation. They find this even more remarkable because parents often cannot make a distinction between a slightly elevated body temperature and fever and seem to overestimate the significance of an elevated body temperature as a single symptom. Nevertheless, parents tend to take the child’s temperature multiple times a day.

*They talk about fever but often parents already call us when the temperature is 37.8 °C or 37.9 °C because they start to worry (Nurse 4,FG1).*

According to WCC professionals parents also lack knowledge regarding pathophysiology and self-management strategies.

*The only thing they think of is, “Fever? Oh I have to give paracetamol!” (…) But not everyone knows you have to go through this and it will pass. And also that if you do nothing, it will pass (Nurse 1, FG2).*

Parents, especially the higher-educated ones, hold strong beliefs that fever needs to be solved and that paracetamol needs to be administrated to lower the body temperature.

*Although I say to them every time not to provide paracetamol. They do it every time. The information just doesn’t stick (Nurse 1, FG1).*

#### Level of experience

WCC professionals perceive that parental worries towards childhood fever are closely related to and driven by parents’ experience of caring for feverish children. That experience is mainly determined by the number and age of children in the family and previous personal experiences with fever-related illnesses. WCC professionals receive most questions from first-time parents with no previous experience with illness in their young child.

*You have to get to know your baby. So it is more to deal with the worries of the parents than with seeing the child (Nurse 1, FG1).*

According to WCC professionals parents gain experience and associated confidence in coping with fever within the course of taking care of one child. Therefore, the age of the child also influences the level of anxiety over fever in parents, according to them.

*The first time is fierce, and the second time too but they don’t call us that quickly anymore when it happens for the third or fourth time (Nurse 3, FG3).*

Gaining experience was also felt to be closely related to gaining more knowledge on how to cope with fever episodes. The WCC professionals believe that this is why they receive fewer questions from parents with multiple children.

The level of parental worries may also be influenced by previous experiences with fever episodes. Specifically, WCC professionals stated that parental worries over childhood fever increase exponentially if parents had a bad experience with a feverish child in their social network in the past:

*If parents have seen how a child ended up in a hospital or experienced a serious condition, it plays a role when their own child starts to get ill (Doctor 1, FG3).*

#### Influence of educational level and social network

WCC professionals suggest a difference in parental attitude towards coping with fever between lower- and higher-educated parents, which in turn might influence the level of worries and frequency of asking questions. According to them, higher-educated parents tend to panic more quickly, act less intuitively and rely more on the Internet and the expertise of WCC professionals than lower-educated parents:

*In my opinion the group of higher-educated parents has a lot of questions. They do not act on their intuition but want to know all the ins and outs, and are unable to let it go (Doctor 3, FG4).*

Participants provided potential explanations for this difference. They indicated that higher-educated parents may tend to ask more questions about fever since an ill child disturbs their work rhythm and may prevent access to child daycare. Higher-educated parents may therefore have a stronger tendency to administer paracetamol to their feverish child.

*We have to see this more from a practical point of view: if a child doesn’t get a fever, it can go to child daycare; otherwise you have to stay home from work (Doctor 2, FG1).*

Participants also indicated that the size of the social network as another explanation for this difference. According to them, higher-educated parents tend to have a smaller social network than lower-educated parents to rely on during childhood illness

*(..)If you have grandparents who support those parents....but if you have a small network like higher-educated parents, I think they are. [Nurse 2,FG 3]: they are very worried (Doctor 1, FG3).*

Participants explained that a substantial social network may provide support by transferring knowledge and sharing experiences regarding fever. However, WCC professionals also acknowledge that the social network may boost parental worries by sharing horror stories on severe underlying illness and/or adverse events and cause more worries among parents.

#### Inconsistent information provision

WCC professionals stated that parental worries and the urge to ask questions may also arise from inconsistent information provision among different healthcare providers or from information sources such as the Internet.

WCC professionals experience differences in paracetamol administration recommendations between themselves and GPs, medical specialists, and chemists. This inconsistency causes worries, confusion and uncertainty about fever management among parents.

*And then they go to the chemist or GP and hear “No, that’s (paracetamol dosage) really not allowed”. “You gave us the wrong advice!” While it was according to our (WCC) guidelines. (Nurse 6, FG2).*

WCC professionals find this difficult and try to comfort parents by explaining the differences.

WCC professionals also observe that it is getting more and more common for parents to search the Internet for information when their child has a fever. However, reading information on the internet may increase parental anxiety and cause parents to contact WCC professionals to check if the information is accurate.

*Parents use Google a lot these days and: “Well the GP told me this but I started searching on the Internet and I read something completely different!” (Nurse 3, FG3).*

#### Suggestions for improvement

WCC professionals stated that currently available written information on fever aimed at parents is very limited and focuses mainly on fever as a possible side-effect of vaccination jabs. More specifically, the discussions revealed a need to better inform parents. WCC professionals believe that to achieve this, it is necessary to meet certain conditions when developing a new information tool.

*Most important is that parents know where they can find the information whenever they need it. Whether they call us, whether it is described in a booklet, whether they can look it up on the Internet. If they just know where to find the most recent information* (Doctor 2, FG1).

The information should be easy to find and written in languages most frequently spoken. WCC professionals prefer a hard copy information tool with visual information to underpin their verbal information. They also want to complement the hard copy information with an accessible and readable website or mobile application. The content of the new information tool should be consistent among healthcare professionals to avoid confusion among parents. The moment at which the tool should be provided to parents was under debate and ranged from antenatal classes to prior to the first vaccination jab when the child is two months old.

*The WCC check-up at 4 weeks is a good moment. Just after the final visit of the midwife, when she (midwife) has already started talking about fever, what of course, she is going to do from now on (Doctor 2, FG4)*

## Discussion and Conclusion

### Discussion

According to WCC professionals parental worries about childhood fever are the major driving factor behind most fever-related contacts with the WCC. Four subcategories were identified as possible drivers of parental worries: level of knowledge, level of experience, influence of educational level and social network, and perceived inconsistency in paracetamol administration advice among healthcare professionals. In addition, WCC professionals believe that current information provision on fever is limited and focuses mainly on fever as a side-effect of vaccination jabs. WCC professionals subsequently expressed the need to improve current information provision and provided starting points in terms of its findability, language, lay-out, content and timing. It was especially mentioned that the content of information provided should become more consistent among different healthcare providers to avoid worries caused by uncertainty. The timing of information provision was under debate but leaned towards the first two months of a child’s life.

According to WCC professional parents often seem to overestimate the significance of body temperature and perceive that the degree of fever indicates the severity of the underlying illness. This finding is in accordance with previous research that parental worries may arise because of the belief that fever is a disease and not just a symptom of illness. Subsequently, when parents view fever as a disease on its own, this will ultimately lead to misconceptions about its role in illness [4, 5]. It is also demonstrated that parental worries lead to the increased use of healthcare services [1, 2, 38].

Corresponding with earlier research, WCC professionals perceived they received most questions from first-time parents, with younger children causing more worries, because those parents lack own experience of coping with fever. As a consequence, parents consult a GP more often for a firstborn child than for a second or subsequent child [39, 40]. Our study also confirms that parents feel uncertain about their actions during fever episodes and consult WCC professionals regularly for advice [4, 10, 21, 40–44].

In line with previous research WCC professionals stated that the social network is an important source of information for parents [10, 40, 45]. WCC professionals experienced that higher-educated parents worry more quickly and tend to rely more on advice of healthcare providers and the Internet than lower-educated parents. It is known from the literature that healthcare professionals and the Internet are an important source of information for parents [4, 10, 40, 43, 45]. Previous research also showed that the Internet is mostly used as information source by younger parents and children [10]. WCC professionals explained the difference between higher and lower educated parents in relying on healthcare professionals by the observation that higher-educated parents seem to have a smaller social network in close proximity to rely on for advice. This observation is in accordance with previous research which demonstrated that parents who did not graduate from high school were less likely to consult a healthcare professional and depended more on nonmedical individuals for advice. However, a lower educational level was also associated with practices that could delay care [15, 46, 47]. Differences among lower and higher educated parents in relying on the social network and healthcare professionals as important information sources should be considered when developing information provision about childhood fever.

Other explanations of why higher educated parents tend to rely more on healthcare providers may lie within the fact that education enhances parents’ knowledge of fever, healthcare facilities and may improve their capability to communicate with healthcare providers [48]. Also, it may be possible that parents with a low income may tend to wait longer to avoid medical expenditures [49]. It was perceived that inconsistency in received advice, due to the use of different guidelines by WCC professionals, GPs, GPs, medical specialists, and chemists, led to confusion, more uncertainty and worries during fever episodes. Previous research already indicated that practice variations exist in treating febrile infants among pediatric emergency physicians [50]. Different studies also showed that providing conflicting information on fever management increases worries among participants, especially when the information comes from sources considered reliable and trustworthy, such as a healthcare professional [12]. Like recent qualitative research among parents presenting to GP out-of-hours services with a febrile child, WCC professionals recommended providing consistent information among different healthcare providers [51].

WCC professionals perceive that the information currently available on fever is very limited, differs across healthcare providers and does not focus on fever as a separate topic but mainly on fever as a possible side-effect of vaccination jabs. In addition, they stated that current information provision on fever needs improvement. Previous research indicates that educational interventions seem to be most effective when they are provided in personal discussions to tailor information to needs, beliefs, experience, and skills, of end-users. In addition, information resources should be accurate, consistent, written, simple to use, and contain simple symbols, [9, 41, 52, 53]. According to Cabral et al [54] interventions may be more effective if they focus on reducing uncertainty in situations when a consultation or antibiotic prescription is needed by increasing knowledge among parents and clinicians about which symptoms need medical attention.

This is the first qualitative study to explore the experiences of WCC professionals towards childhood fever and current information provision, to inform future interventions aimed at educating parents prior to their child’s first fever episode. A strength of our study is the inclusion of WCC professionals working at different WCC locations in the region of Maastricht (the Netherlands), thereby including neighbourhoods with a variety of socioeconomic statuses. We achieved methodological and investigator triangulation, held peer debriefings with the wider research team and a member check.

Dutch WCC has specially trained doctors and nurses in preventive youth healthcare, while GPs have a gatekeeping role and mostly focus on curative youth healthcare. The tasks of Dutch WCCs do resemble the tasks of other preventive healthcare services worldwide We believe that our findings regarding information provision about childhood fever may be transferable to other countries as well.

We purposefully sampled WCC professionals from a small and deprived region in the Netherlands with a limited diversity in ethnicities. It is therefore possible that the views and experiences may differ from WCC professionals in other regions. It is important to keep in mind that the WCC professionals expressed their thoughts about possible drivers of fever-related contacts in parents and we did not investigate the experiences of parents themselves.

## Conclusions

Fever-related questions are common in well-child care and professionals perceive that most of the workload is driven by parental worries. Four categories were identified as possible drivers of parental worries: lack of knowledge, lack of experience or having had negative experiences with fever in the past, educational level and size of social network and inconsistencies in paracetamol administration advice. This study demonstrates that current information provision on fever is limited and current information provision need to be optimised and should acknowledge patients’ characteristics and inconsistencies among healthcare providers.

Based on these results we can state that future interventions should aim to lower worries among parents. It is important not only to educate parents on an ad hoc basis when children are ill but also prior to a child’s first fever episode to prepare parents for future illness. This can be achieved by tailoring interventions to the needs of parents and accounting for their level of knowledge, experience, educational level and availability of a social network. To clarify, future fever information provision should focus on improving fever management and practical skills since parents seem to lack knowledge of fever pathophysiology and self-management strategies. Particular attention should be paid to first-time parents who lack experience with childhood fever episodes and to higher-educated parents with a small social network. It is also essential that paracetamol administration advice is consistent among different healthcare professionals. Furthermore, information should be easy to find, easy to understand and verbal information provision needs to be supported by hard copy visual information and web-based applications. The timing of information provision on fever is still under debate but the tendency is to provide it within the first two months of a child’s life.

## Abbreviations

FG, Focus group discussion; GP, general practitioner; WCC, well-child clinics

## Additional file

Additional file 1: Topic list used for focus group discussion 1 and 4. (DOCX 30 kb)
