# Supplementary material for: Childhood fever in well-child clinics: a focus group study among doctors and nurses
Source: BMC Health Serv Res. 2016 Jul 8;16:240. doi: 10.1186/s12913-016-1488-1 (PMC4938983; doi:10.1186/s12913-016-1488-1)
Supplement: Additional file 1: — Topic list used for focus group discussion 1 and 4. (DOCX 30 kb) [file 12913_2016_1488_MOESM1_ESM.docx]

**Topic list of first focus group discussion**Focus group discussions are held with doctors and nurses working at well-child clinics providing preventive youth health care services to children aged 0-4 years old.

| **Theme 1.**  **Experiences and current practices.** |
| --- |
| What are your experiences with childhood fever at the well-child clinic? |
| What kind of questions do you receive from parents regarding fever? |
| When do you receive questions from parents about fever: Prior to childhood illness to prepare for new episodes? Or when children are ill? |
| How do you cope with fever-related questions from parents? |
|  |
| **Theme 2.**  **The need to improve information provision about fever.** |
| What do you think of the current information provision about fever at the well-child clinic? |
| Can you provide us with a detailed description of the way you provide information to parents about childhood fever? |
| What is going well in the current information provision about fever? |
| Do you experience barriers in the current information provision on fever? |
| What do you miss in the current information provision about fever? |
| Do you have any ideas on how to improve the current information provision on childhood fever? |

**Topic list final focus group discussion**

| **Theme 1.**  **Experiences and current practices.** |
| --- |
| What are your experiences with childhood fever at the well-child clinic? |
| What kind of questions do you receive from parents regarding fever? |
| From who do you receive most questions? |
| When do you receive questions from parents about fever: Prior to childhood illness to prepare for new episodes? Or when children are ill? |
| How do you cope with fever-related questions from parents? |
| Is the content or method of information provision on fever, tailored to differences among parents in terms of educational level or cultural background? |
|  |
| **Theme 2.**  **The need to improve information provision about fever.** |
| What is your opinion about the current information provision on childhood fever at the well-child clinic? |
| Can you provide us with a detailed description of the way you provide information to parents about childhood fever? |
| What is going well in the current information provision about fever? |
| Do you experience barriers in the current information provision on childhood fever? |
| What do you miss in the current information provision about fever? |
| Do you have any ideas on how to improve the current information provision on childhood fever? |
| How would you prefer to provide information to parents about fever? |
